# Reduction in Powder Wall Friction by an a-C:H:Si Film

**DOI:** 10.3390/ma17102421

**Published:** 2024-05-17

**Authors:** Christof Lanzerstorfer, Christian Forsich, Francisco Delfin, Manuel C. J. Schachinger, Daniel Heim

**Affiliations:** 1 School of Engineering, University of Applied Sciences Upper Austria, Stelzhamerstraße 23, 4600 Wels, Austria; christian.forsich@fh-wels.at (C.F.); francisco.delfin@fh-wels.at (F.D.); manuel.schachinger@fh-wels.at (M.C.J.S.); daniel.heim@fh-wels.at (D.H.); 2 Regional Faculty of Concepción del Uruguay (UTN-FRCU), National University of Technology, Ing. Pereyra 676, Concepción del Uruguay 3260, Argentina

**Keywords:** powder, wall friction, diamond-like carbon

## Abstract

The wall friction angle is an important parameter in powder flow. In a recent study for various powders, a reduction in the wall friction angle for steel was demonstrated by the application of an a-C:H:Si film on the steel surface. This work presents the results of a study of this effect in more detail regarding the influence of the powder material, the wall normal stress and the particle size of the powder for mass median diameters from 4 µm to approximately 150 µm. The wall friction angles were measured using a Schulze ring shear tester for three different powder materials: aluminum oxide, calcium carbonate and silicon carbide. The results showed little difference with respect to powder chemistry. For the coarser powders, the reduction in the wall friction angle due to the a-C:H:Si coating was highest (10° to 12°) and rather stress-independent, while for the fine and medium-size powders the reduction was lower and stress-dependent. With increasing wall normal stress, the reduction in the wall friction angle increased. These results can be explained by the friction reduction mechanism of a-C:H:Si, which requires a certain contact pressure for superficial graphitization.

## 1. Introduction

In the design of particulate material handling and conveying equipment, friction between the particulate material and the wall material plays a crucial role. The friction between the powder and the wall material can be described using the wall friction angle φ_W_ or the wall friction coefficient µ [1]. The wall friction coefficient is the ratio of the wall shear stress τ_W_ to the wall normal stress σ_W_ and the wall friction angle is the arctangent of the wall friction coefficient. Powder wall friction depends on both the powder properties and the wall material. The particle size of the powder is an important parameter with respect to wall friction. Lower values of the wall friction angle for granular material with larger particle size were reported for fine granular materials, like wet gypsum [2], and for fly ash from biomass combustion plants [3] as well as for steel mill dedusting residues [4]. In contrast, for large particles with a diameter of more than several hundred micrometers an increase in wall friction with the particles size was reported [5]. Another important parameter for wall friction is the moisture content of the powder. For various food powders, the wall friction angle increased with the moisture content [6]. However, it is not always certain whether an increased moisture content of the powder will increase or decrease the wall friction angle [2,5]. Further powder properties determined to influence wall friction are the particle size distribution, the surface structure of the particles, the particle hardness and the chemical composition [1].

The most important property of the wall material with respect to wall friction is the surface roughness [7]. With increasing roughness of the wall material, increasing wall friction angles were reported for salt [8], for limestone powder [9] and for polyethylene pellets [10]. Other parameters of the wall material that influence wall friction are its hardness [11] and its chemical composition [1]. 

The outstanding properties of diamond-like carbon (DLC), such as a high degree of hardness and chemical inertness, are two of the reasons for the wide use of DLC-based films in different applications. DLC-based films possess very good tribological properties such as low friction coefficients and low wear rates [12]. Additionally, the material is very corrosion-resistant and biologically compatible [13]. Therefore, DLC-based coatings find widespread use for friction and wear reduction on sliding mechanical parts [14,15]. To further improve the already beneficial properties of DLC, silicon doping may be applied as silicon only forms tetrahedral (sp^3^-type) bonding and thus increases the hardness of the amorphous carbon [16,17].

The low friction coefficient of DLC-based coatings is explained by graphitization of the DLC, where the formed debris is acting as a third body, and by the formation of a transfer film onto the counterpart. These processes need the transfer of energy into the contact zone and depend on the sliding speed and sliding distance and also on the contact pressure [18,19,20,21].

While the benefit of DLC-based coatings in friction reduction for sliding and rotating parts has been investigated widely [22,23,24,25,26], the use of DLC-based coatings for powder wall friction reduction has been suggested only recently [27]. However, in the literature search, no further publications were found on the topic of powder wall friction reduction through DLC-based coatings. In [27], a reduction in the wall friction angle by the a-C:H:Si film of 2°–14° was reported in comparison with a stainless steel material of similar surface roughness. The level of reduction was not constant but depended on the type of powder material, as well as on the wall normal stress. 

The aim of the present study was to investigate the reduction in wall friction for various powders by employing an a-C:H:Si film and determine the influencing parameters. The wall friction angle of three different materials, aluminum oxide, calcium carbonate and silicon carbide, with three different mass median diameters in the range of 4.0 µm to approximately 150 µm for each material was measured for a-C:H:Si-coated stainless steel, uncoated stainless steel with a similar roughness and an ultra-high molecular weight polyethylene (UHMWPE) material with a somewhat higher wall roughness.

## 2. Materials and Methods

The a-C:H:Si film was deposited on a stainless-steel sample (EN 1.4301) in a commercial hot wall plasma-assisted chemical vapor deposition (PACVD) reactor manufactured by Rübig GmbH & Co KG (Wels, Austria). The a-C:H:Si film produced consisted of an amorphous hydrogenated carbon film modified by embedded Si in the carbon matrix. Silicon was chosen as a dopant to increase the hardness and thus the wear resistance of the film. The reactor vessel of the PACVD reactor has an inner diameter of 400 mm and a height of 600 mm, which was evacuated to a base pressure of 1 Pa using a screw pump and a roots blower controlled by a throttle valve. A pulsed DC voltage of 350 V, with a frequency of 1.5 kHz and a duty cycle of 25% was applied to the reactor in such way that the substrate plate acted as cathode and the wall of the chamber acted as anode. The gas mixture consisted of 15% acetylene and 1% hexamethyldisiloxane (HMDSO), as carbon and silicon precursors, respectively, with argon completing the gas mixture, under an operating pressure of 200 Pa. The process temperature was set to 450 °C, which was measured by thermocouples inserted in the core of dummy samples placed on the deposition plate. Prior to the main process, a thin silicon-based interlayer was deposited to improve adhesion and performance of the coating. Deposition time was 35 h, with an estimated deposition rate of about 1 µm/h. Further information concerning the PACVD system has been published previously [28].

Powder samples of aluminum oxide, calcium carbonate and silicon carbide were used in the wall friction tests. The three powder materials differ significantly in terms of hardness. While the Mohs hardness of calcium carbonate is 3 the Mohs hardness of aluminum oxide and silicon carbide is 9 and 9.5, respectively. From each powder material, aluminum oxide, calcium carbonate and silicon carbide, three samples with different particle sizes were used: (1) fine powders with a mass median diameter d_50_ of approximately 4 µm; (2) medium-size powders with a d_50_ in the range of 14 to 32 µm; and (3) coarse powders with a d_50_ of approximately 150 µm. The fine silicon carbide is F 1200-D and the medium-size silicon carbide is P 600, both from ESK-SiC GmbH (Frechen, Germany). The coarse silicon carbide is 100 mesh silicon carbide from abcr GmbH (Karlsruhe, Germany). The fine calcium carbonate is the certified reference material BCR-116 purchased from the Institute for Reference Materials and Measurements of the European Commission for shear tests. The medium-size calcium carbonate is calcium carbonate Normapur from VWR International (Radnor, PA, USA) and the coarse calcium carbonate was made by sieving crushed limestone using 160 µm and 63 µm sieves. The size fraction 63–160 µm was used as the coarse calcium carbonate. The fine aluminum oxide and the medium-size aluminum oxide were obtained from KUVAG GmbH (Neumarkt, Austria) without a specification and the coarse aluminum oxide is fused brown aluminum oxide for sandblasting from Washington Mills.

For the measurement of the wall friction angle of the particulate materials, an RST-XS ring shear tester from Schulze was used. In this test, a sample of the wall material forms the bottom ring of the wall friction shear cell. The measuring method is described in detail in the standard ASTM D6773 [29]. In addition to the wall material with the a-C:H:Si film, a wall sample of uncoated stainless steel EN 1.4301 and a sample of UHMWPE were used in the tests. 

The wall yield locus results from corresponding values of the normal stress and the shear stress. The kinematic angle of wall friction is the slope of a straight line running through the origin and a point of the wall yield locus [1]. In the tests, each measurement was repeated five times. In the results, the average wall friction angle and its standard deviation are shown. The measurements were performed using the whole measuring range of the shear tester. The used values of wall normal stress (240 Pa, 600 Pa, 2000 Pa, 6000 Pa and 20,000 Pa) are approximately equidistant on a logarithmic scale. 

The particle size distribution of the powder samples was measured by laser diffraction using a HELOS/RODOS instrument from Sympatec GmbH (Clausthal-Zellerfeld, Germany) with dry sample dispersion. The calibration of the instrument was checked with a Sympatec SiC-P600′06 reference material. Additionally, microscopic images of the various powder samples were taken with a TESCAN (Brno, Czech Republic) scanning electron microscope (SEM), type MIRA3.

The moisture content of the powders was determined by drying at 105 °C before and after the measurement of the wall friction angle. The surface roughness of the wall samples was measured along two perpendicular axes with a surface roughness tester from Mitutoyo (Kawasaki, Japan), type SJ-201p. The instrument scans the unevenness of the surface with a measuring feeler. The vertical displacement of the feeler pin is recorded and converted to the standardized output values R_a_ (arithmetical mean roughness value) and R_z_ (mean roughness depth). The Nano Indenter XP measurement system from MTS was used to measure the hardness of the a-C:H:Si film and the stainless steel sample. The measurements were carried out at a maximum indentation load of 30 mN using a Berkovich-type diamond indenter. The hardness of the UHMWPE sample was measured with a Shore durometer from Zwick Roell (Ulm, Germany).

Further characterization of the a-C:H:Si coating was carried out using SEM (TESCAN MIRA 3) coupled with EDX analysis (Oxford AZtecEnergy XT, Oxford Instruments, Abingdon, UK) to examine the surface morphology as well as the elemental composition of the DLC. In addition, Raman spectroscopy (Horiba XploRA Plus, Kyoto, Japan), equipped with a 532 nm laser, was used to analyze the structure and bonding of the carbon material. The obtained Raman spectra, showing the characteristic D and G bands, were subsequently fitted by Gaussian–Lorentzian functions to obtain relevant Raman parameters such as the position and FWHM of the G band, the I(D)/I(G) ratio and the baseline slope of the spectrum. 

## 3. Results

### 3.1. Powder Samples

The measured data of the powder samples used in the tests are summarized in Table 1. The humidity of all samples was very low and did not change during the wall friction measurements. 

**Figure 1:**
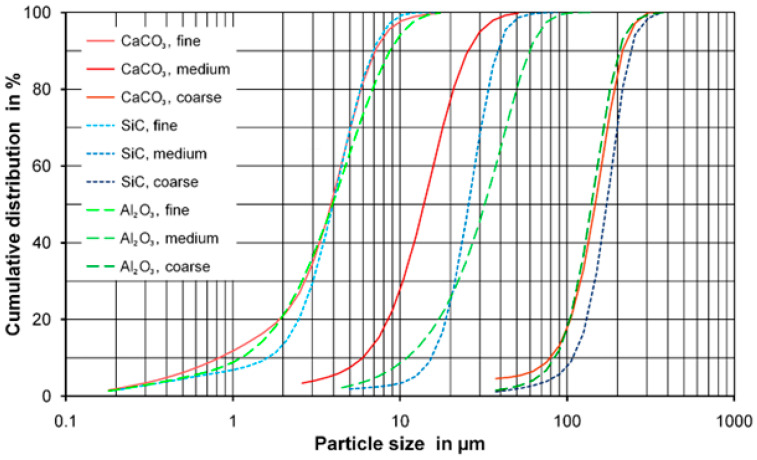
Cumulative size distribution of the aluminum oxide, calcium carbonate and silicon carbide powder samples.

Figure 1 shows the particle size distribution of the aluminum oxide, calcium carbonate and silicon carbide powders. The size distributions of the three different samples of the fine powder and the three coarse powders were very similar, while there were some differences in the size distributions of the medium-size powder samples.

**Figure 2:**
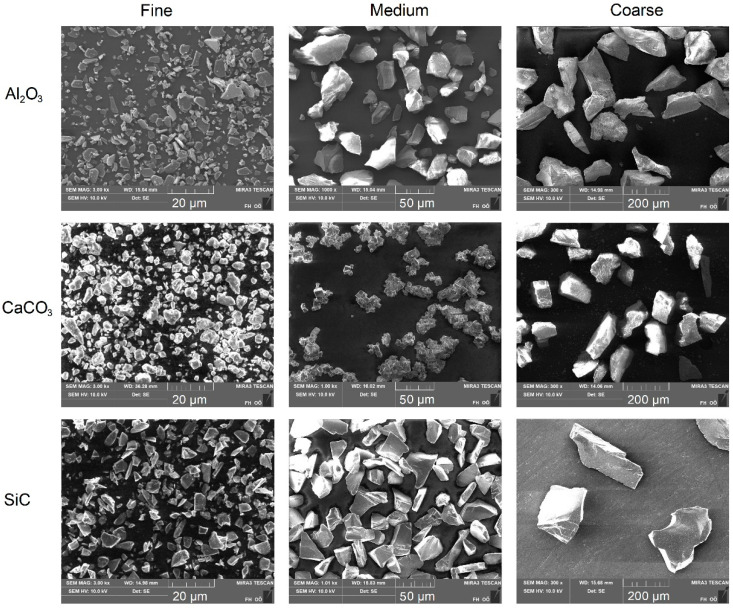
Microscopic images of the aluminum oxide, calcium carbonate and silicon carbide powder samples.

Microscopic images of the various aluminum oxide, calcium carbonate and silicon carbide powder samples are shown in Figure 2. All samples consisted mainly of angular and relatively compact particles.

### 3.2. Wall Samples

The values of the mean roughness depth R_z_ and the arithmetical mean roughness value R_a_ were quite similar for the wall sample with the a-C:H:Si film and the uncoated 1.4301 sample, whereas the values for the UHMWPE material were approximately twice as high. The hardness of the a-C:H:Si film was approximately seven times higher compared to the hardness of the uncoated 1.4301 material. The measured results of the surface roughness and the hardness are summarized in Table 2. 

**Figure 3:**
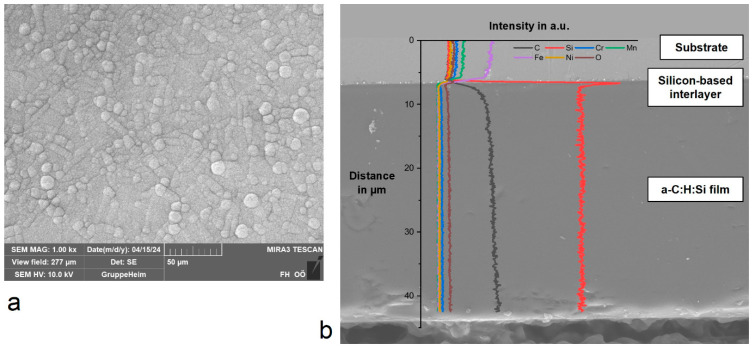
(**a**) Scanning electron microscope image showing the morphology of the a-C:H:Si film; (**b**) EDX line-scan of the cross-section of the a-C:H:Si film.

Surface characterization via SEM, showing the characteristic globular morphology, and an EDX line-scan of the cross-section of the a-C:H:Si film with the chemical composition as a function of the distance are shown in Figure 3. The different areas of the cross-section correspond to the substrate material, the silicon-based interlayer as well as the a-C:H:Si film. The line-scan further demonstrates the homogenous distribution of silicon inside the a-C:H:Si along the cross-section, with an average silicon content of 15.7 at.-% according to EDX. 

**Figure 4:**
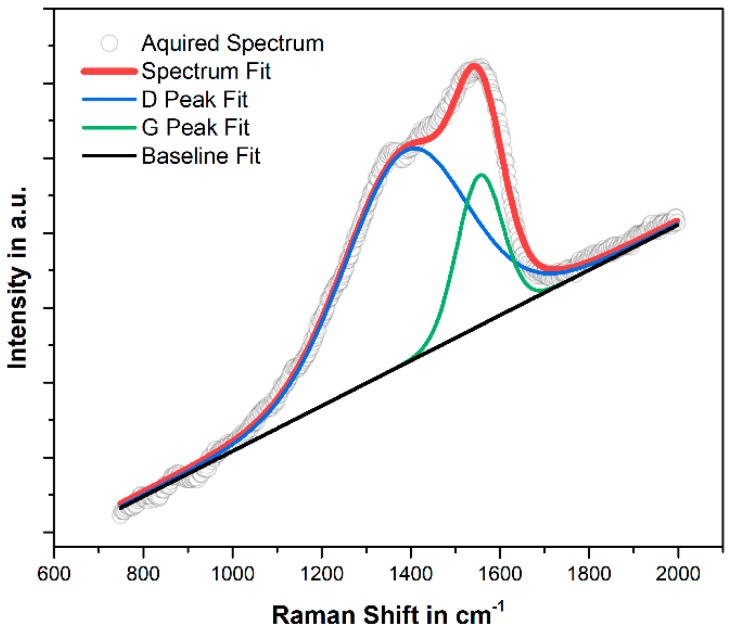
Deconvoluted Raman spectrum of the a-C:H:Si coating, obtained using a 532 nm excitation wavelength.

Typically, carbon Raman bands manifest at 1580 cm^−1^ and 1350 cm^−1^ corresponding to the G and D bands, respectively. However, in the acquired spectrum of the a-C:H:Si shown in Figure 4, these peaks exhibit a notable shift towards the center of the spectrum, with the G band positioned at 1555 cm^−1^ and the D band at 1389 cm^−1^. The lower position of the G band, along with its broadening (FWHM(G) = 120 cm^−1^) indicate high topological as well as structural disorder, the latter resembling the distortion of bonding lengths and angles of the carbon network [30]. The increase in structural and in topological disorder may both be partly attributed to the incorporation of Si atoms into the amorphous carbon as silicon exhibits a larger atomic radius than carbon and only forms tetrahedral (Si-C sp^3^) bonding, ultimately distorting the amorphous carbon network [17]. 

Even further, the hydrogen content estimated with the baseline slope was about 41%, according to the method described in [31]. The downward shift of the G band, alongside with the significant degree of structural disorder present in the material, suggests a high fraction of total sp^3^ hybridization. However, considering the substantial presence of hydrogen due to the HMDSO doping, which terminates the C-C sp^3^-bonded carbon backbone as well as the Si-C sp^3^ bonding, lead to the conclusion that the number of diamond-like C-C sp^3^ bonds and Si-C sp^3^ bonds relative to the total fraction of sp^3^ bonding is relatively low. This is consistent with the reduced hardness values of the obtained a-C:H:Si in comparison to other DLC films, as hardness is mainly a function of tetrahedral carbon bonding [32].

### 3.3. Particle Size versus Roughness of Wall Material

According to the literature [33], the ratio of particle size to wall material roughness can be used to characterize the wall friction because particles smaller than the roughness depth in contact with the wall can become trapped in grooves. With a substantial fraction of particles smaller than the mean roughness depth of the wall material, a significant proportion of the movement of the bulk of the powder relative to the wall takes place by means of flow within the powder [34]. This results in an apparent increase in wall friction, because the angle of internal friction of a powder is usually much higher than the wall friction angle between the particles and the wall material [7].

For the fine aluminum oxide, calcium carbonate and silicon carbide powders the fraction of the volume of the powder which is smaller to the mean roughness depth of the wall material was in the range of approximately 30% for the a-C:H:Si film and the 1.4301 material to approximately 75% for the UHMWPE wall material. For the medium-size powders, it was less than 5% for all wall materials and powders except when pairing calcium carbonate with UHMWPE wall material, where the fraction was approximately 10%. For the coarse powder samples, the fractions smaller than the mean roughness depth of the wall material were even smaller.

### 3.4. Particle Hardness versus Hardness of Wall Material

The Mohs hardness of calcium carbonate of 3 corresponds to approximately 1.3 GPa, the Mohs hardness of aluminum oxide and silicon carbide of 9 and 9.5 corresponds to 20 GPa and 27 GPa, respectively [35]. Consequently, the hardness of aluminum oxide and silicon carbide was much greater than the hardness of each of the wall materials. By contrast, the hardness of calcium carbonate was similar to that of the stainless steel 1.4301 wall material and, therefore, markedly lower than the hardness of the a-C:H:Si film.

### 3.5. Wall Friction Angles

**Figure 5:**
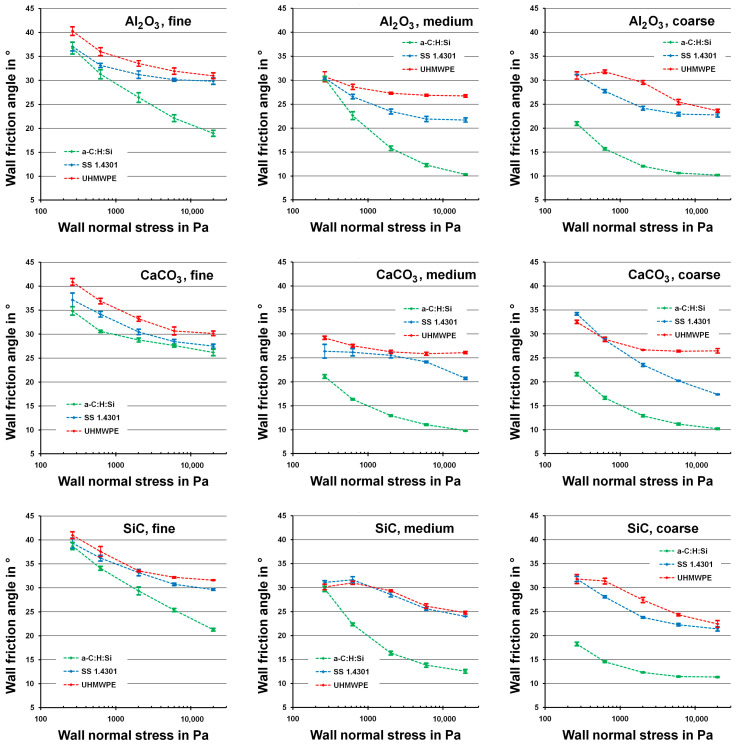
Wall friction angle as a function of the wall normal stress for the various aluminum oxide, calcium carbonate and silicon carbide powder samples.

Figure 5 shows the wall friction angle of the various aluminum oxide, calcium carbonate and silicon carbide powder samples with the a-C:H:Si film and the two other wall materials in dependence of the wall normal stress. Generally, the reproducibility of the measurements was very good. The relative standard deviation of the measured wall friction angles was in the range of 1% to 4%. 

The wall friction angles decrease with increasing wall normal stress, which is in accordance with various published data [36,37,38,39]. However, this effect was not equally pronounced for all wall materials and powder particle sizes. 

#### 3.5.1. Influence of the Wall Material on the Wall Friction Angle

The wall material significantly influenced the wall friction angle. In all measurements, the wall friction angles with the a-C:H:Si-coated material were smaller than the wall friction angles with stainless steel 1.4301. The wall friction angles with UHMWPE were a few degrees larger than with stainless steel 1.4301 in all cases, except for the coarse powders at the lowest value of the wall normal stress.

The observed reduction in wall friction by the a-C:H:Si film was not constant. It was usually lower for smaller values of the wall normal stress and for smaller particles.

#### 3.5.2. Influence of the Particle Size on the Wall Friction Angle

**Figure 6:**
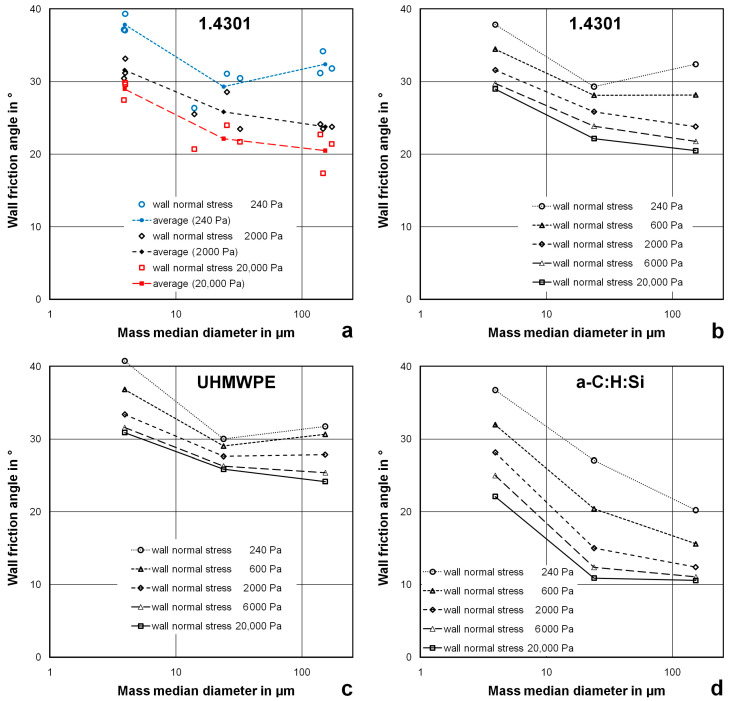
Dependence of the wall friction angle on the particle size; (**a**) wall material 1.4301, data points for different powder materials; (**b**–**d**) average values for fine, medium-size and coarse powder materials.

Figure 6 shows the dependence of the wall friction angle on the particle size. The wall friction angles with the stainless steel 1.4301 material were higher for the fine powders and lower for the medium-size and the coarse powders. Higher values of the wall friction angle for very fine powders are in accordance with the findings from other studies [2,3,4]. Since the influence of the material of the powder was not very high (Figure 6a) average values were calculated for the different powder materials for the various levels of wall normal stress (Figure 6b). Independent of the wall normal stress, the wall friction angle was approximately 5° smaller for the medium-size powders compared to the fine powders. For the coarse powders, the wall friction angle was even slightly lower at high values of the wall normal stress, had the same value for medium values of the wall normal stress and was somewhat higher for low values of the wall normal stress. For the UHMWPE wall material, similar trends were found with slightly higher values of the wall friction angle (Figure 6c).

The dependence between particle size and wall friction angle for the a-C:H:Si film was significantly different (Figure 6d). Firstly, the wall friction angle’s dependence on the wall normal stress was more pronounced for the fine and medium-size powders. Secondly, the reduction in wall friction for the fine particle size and the medium particle size was higher, and, thirdly, there was a further reduction in the wall friction for the medium particle size and the large particle size. In contrast to stainless steel 1.4301 and UHMWPE, this further reduction was especially high at lower values of the wall friction angle. 

#### 3.5.3. Influence of the Powder Material on the Wall Friction Angle

The measured wall friction angles for the very hard powder materials of aluminum oxide and silicon carbide were very similar, while for the softer calcium carbonate, slightly different results were obtained (Figure 5). However, the influence of the powder material on the wall friction angle was rather small compared to the influence of the wall material and the particle size of the powder. 

## 4. Discussion

**Figure 7:**
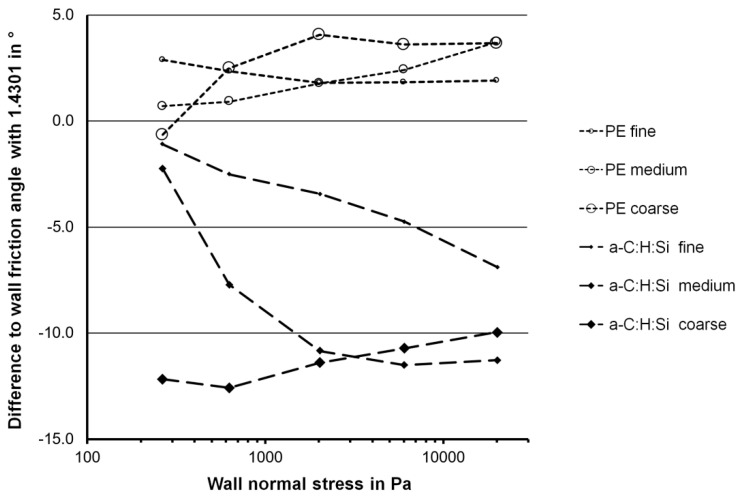

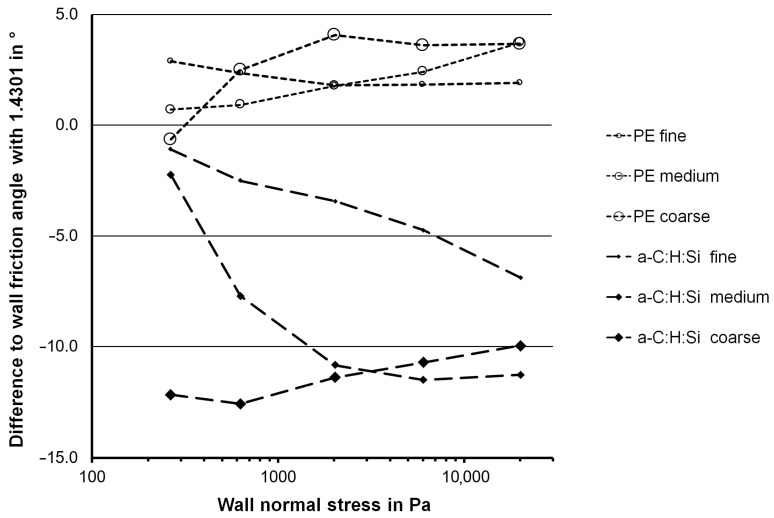
Differences between the average wall friction angles for the specified wall material to the average wall friction angle for 1.4301 for the three powder sizes (fine, medium and coarse) as a function of the wall normal stress.

The biggest influence on the wall friction angle was found to be the wall material. A significant reduction in wall friction for the a-C:H:Si film was observed in comparison to stainless steel 1.4301, while in most cases the wall friction angle for UHMWPE was somewhat higher than with stainless steel 1.4301. Because of the small influence of the powder material, average wall friction angles were calculated for the three materials. Figure 7 shows the differences between the average wall friction angle for the a-C:H:Si film and with the UHMWPE material to the average wall friction angle for the stainless steel 1.4301 as a function of the wall normal stress. The average wall friction angles with UHMWPE were, with one exception, 1° to 4° degrees larger than with 1.4301. This difference was almost independent of the wall normal stress. The higher wall friction angles for UHMWPE might be partly explained by the higher surface roughness values of this material. 

In all measurements, the wall friction angle for the a-C:H:Si-coated material was lower than the wall friction angle for stainless steel 1.4301. For the coarse powders, the reduction in the wall friction angle was 12° at a wall normal stress of 240 Pa, the lowest value of the wall normal stress measured. At higher values of the wall normal stress, the reduction decreases steadily to 10°. This is a result of the fact, that the wall friction angle for the a-C:H:Si-coated material gradually approaches a value of approximately 10°, while the wall friction angle for the stainless steel 1.4301 decreases further.

For the fine powders, at low values of the wall normal stress the reduction in wall friction was very small. However, the reduction increased steadily with increasing wall normal stress and reached approximately 6° at a wall normal stress of 20,000 Pa. 

For the medium-size powders, the wall friction reduction at low wall normal stress was very small, too, but reached 11° at a wall normal stress of approximately 2000 Pa. At higher values of the wall normal stress the reduction stayed nearly constant at the same level as it was found for the coarse powders.

An explanation of the difference in wall friction reduction might be the differences in the contact stress between the particles and the wall material. The number of contact points per unit area between the wall material and the powders increases from the coarse powders (average d_50_ of approximately 150 µm) to the medium-size powders (average d_50_ of approximately 25 µm) and the fine powders (average d_50_ of approximately 4.0 µm). Parallel to the increase in contact points, the average stress per contact point at a given value of the wall normal stress decreases. 

**Figure 8:**
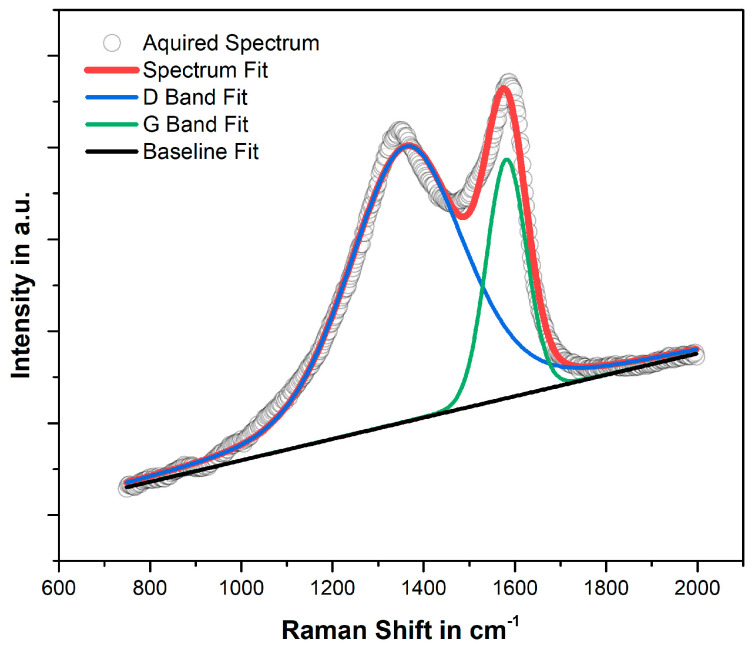
Deconvoluted Raman spectrum of the a-C:H:Si coating showing graphitization, obtained using a 532 nm excitation wavelength.

A higher contact pressure produces an enhancement of the graphitization process of the a-C:H:Si coating, which is ultimately responsible for the low friction coefficient. The Raman spectrum in Figure 8 highlights alterations on the surface of the a-C:H:Si coating compared to its original state as depicted in Figure 4. Notably, the characteristic bands are separated due to a shift of the G band towards higher wavenumbers, specifically from 1555 cm^−1^ to 1581 cm^−1^, which corresponds to a decrease in topological disorder in the film. Moreover, a significant reduction in the FWHM of the G band, from 120 cm^−1^ to 101 cm^−1^, indicates a pronounced decrease in structural disorder [30]. Additionally, a reduction in the estimated hydrogen content from 41% to 34% was observed. All these mechanisms are attributed to the conversion of sp^3^ sites into more ordered graphite-like sp^2^ C-C bonds, i.e., the onset of graphitization, providing an explanation for the phenomenon of the reduction in the wall friction angle during the testing. Furthermore, the steep decrease in topological and structural disorder in the material may also be partly ascribed to the removal of silicon in the a-C:H:Si network due to tribological loading [17]. 

## 5. Conclusions

This study shows that a-C:H:Si coating can reduce wall friction of powders significantly. The effect of the a-C:H:Si film in wall friction reduction was dependent on wall normal stress and particle size, but nearly independent of the powder material. Therefore, the reduction in wall friction angle depends on the contact stress of the particles. For coarser powders, a significant wall friction reduction was found even at low values of the wall normal stress. For finer particles, markedly reduced wall friction angles were found only at somewhat higher values of the wall normal stress. 

## Figures and Tables

**Table 1:** Powder samples.

Particulate Material	Mass Median Diameter d_50_	Moisture
Al_2_O_3_ fine	3.95 µm	0.26%
Al_2_O_3_ medium	32.3 µm	<0.05%
Al_2_O_3_ coarse	140 µm	<0.05%
CaCO_3_ fine	3.87 µm	0.19%
CaCO_3_ medium	14.0 µm	0.15%
CaCO_3_ coarse	147 µm	<0.05%
SiC fine	3.96 µm	<0.05%
SiC medium	25.4 µm	<0.05%
SiC coarse	173 µm	<0.05%

**Table 2:** Wall materials used in the wall friction test.

Wall Material	R_a_ ^2^	R_z_ ^3^	Hardness
Stainless steel 1.4301	0.46 µm	3.3 µm	1.5 GPa
a-C:H:Si film	0.51 µm	3.1 µm	10.2 GPa
UHMWPE	1.04 µm	6.5 µm	65.2 Shore D (0.07 GPa) ^1^

^1^ For better comparison, the hardness of the UHMWPE was also measured with the nanoindentation instrument; ^2^ arithmetical mean roughness value; ^3^ mean roughness depth.

## Data Availability

The raw data supporting the conclusions of this article will be made available by the authors on request.
